# Nevirapine induced Stevens–Johnson syndrome in an HIV infected patient

**DOI:** 10.4103/0253-7613.75680

**Published:** 2011-02

**Authors:** Harminder Singh, Vinay Kumar Kachhap, Bithika Nel Kumar, Kalpana Nayak

**Affiliations:** Department of Pharmacology, Government Medical College, Jagdalpur, Chhattisgarh, India; 1 C.C. Centre, NACO, Department of Medicine, M.P.M. Hospital, Jagdalpur, Chhattisgarh, India

**Keywords:** Adverse drug reaction, highly active antiretroviral therapy, nevirapine, Stevens–Johnson syndrome

## Abstract

Nevirapine, a non-nucleoside reverse transcriptase inhibitor (NNRTI), is widely prescribed as a part of the combination therapy of human immunodeficiency virus (HIV) infection because of its efficacy and good tolerability. Here, we report a case of Stevens–Johnson syndrome (SJS) secondary to nevirapine. The patient had a diffuse, exfoliating exanthema with generalized bullous eruptions that involved the face, trunk and both extremities with elevated hepatic alanine aminotransferase and aspartate aminotransferase enzyme activities. The condition improved with stoppage of nevirapine-based highly active antiretroviral therapy (HAART) regimen, so we attributed this adverse event to nevirapine. A strict vigilance of adverse drug reaction is required in HAART.

## Introduction

Nevirapine, a non-nucleoside reverse transcriptase inhibitor (NNRTI), is widely prescribed as a part of the combination therapy of human immunodeficiency virus (HIV) infection because of its efficacy and good tolerability.[1] It binds directly to reverse transcriptase (RT) and blocks RNA-dependent and DNA-dependent DNA polymerase activity, causing disruption of the enzyme’s catalytic site.

In 1997, nevirapine became the first NNRTI available for the treatment of HIV infection. Its efficacy was established both for the treatment of naive patients and in oversimplification strategies.[2] Nevirapine-based regimens of highly active antiretroviral therapy (HAART) have been widely used in resource-restricted countries because of their efficacy, accessibility and comparatively low cost.[3]

World Health Organization (WHO) recommends nevirapine and efavirenz (EFV) as one of the first-line drugs. The drug is widely available and is less costly than EFV; however, a higher incidence of rash is associated with it than with EFV. The most serious toxic effects associated with nevirapine are skin reactions and liver dysfunction; both are generally mild to moderate and usually an early phenomenon, occurring during the first 6-8 weeks of therapy.[4]

Clinical studies have demonstrated that sustaining viral suppression and maintaining higher CD4 count, mostly as a result of effective combination antiretroviral therapy, delay or prevent some AIDS/non-AIDS–defining complications, such as HIV-associated kidney disease. Sustained viral suppression and immune recovery may also delay or prevent other disorders such as liver disease, cardiovascular disease, malignancies, etc.

Patients infected with human immunodeficiency virus-1 (HIV-1) are at increased risk of developing severe mucocutaneous drug reactions. We report a patient infected with HIV-1 who developed Stevens-Johnson syndrome (SJS) in association with nevirapine. Written informed consent was taken from the patient for collection of her details and images.

## Case Report

A 45-year-old tribal was diagnosed with advanced HIV infection with CD4 cell count of 174 cells/mm^3^ and a viral load of 6800 copies/mL. On the basis of National Aids Control Organization (NACO) guided regimen, stavudine (30 mg), lamivudine (150 mg) and nevirapine (200 mg) were initiated once daily in the month of May 2010 for 15 days, after which she was shifted to twice daily doses. After 3 weeks of starting HAART, she came with complaints of weakness for which she was kept in observation and treated symptomatically and then discharged without any change in HARRT regimen.

**Figure 1:**
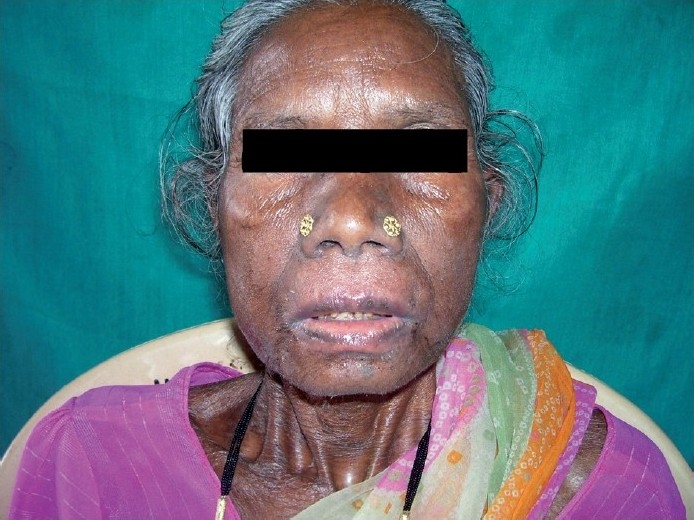
Crust and erosions on the patient’s lips.

The patient was again admitted to Community Care Centre (M.P.M Hospital) affiliated to NACO’s ART centre (Government Medical College, Jagdalpur) with history of oral ulceration for 2 days, nausea, anorexia, slight headache and fever since the previous 24 hours. After 2 days, she developed generalized skin eruption. On examination, she was non-icteric, but had a diffuse, exfoliating exanthema with generalized bullous eruption that involved the trunk, face, and both extremities. In addition, she had facial and lip swelling, oral ulceration and difficulty in opening the mouth and swallowing [Figure 1]. The rash started bilaterally in the arms, then extended to the trunk and the whole body, and was accompanied by generalized pruritus.

At this time, her white blood cell count was 13.8 × 10^9^/L with 63% neutrophils, 22% lymphocytes, 6% monocytes, and 9% eosinophils. Her alanine aminotransferase and aspartate aminotransferase levels were 215 U/L (10-40 U/L) and 117 U/L, (10-35 U/L), respectively. In view of severe skin eruption and impaired hepatic enzymes, the HAART regimen was withheld temporarily.

The patient was treated aggressively with i.v. dexamethasone, 8 mg 6 hourly, i.v. fluids, prophylactic antibiotics, anti-allergic drugs and local treatment of lesions. Stavudine (30 mg), lamivudine (150 mg) and nevirapine (200 mg) regimen was stopped. The patient’s condition improved over the next 4–5 days. The hepatic enzymes remained elevated for 1 week. Re-challenge with nevirapine was never performed but a modified HAART regimen was started that included efavirenz instead of nevirapine, after 15 days of complete resolution of all symptoms and near normalization of hepatic enzymes. No recurrence of rash and impairment of hepatic function were recorded in subsequent follow-up.

## Discussion

In the face of the global AIDS pandemic, advances in its treatment have been strikingly impressive, for example, patients on antiretroviral therapy now live at least 13–14 years longer than those without the therapy.[5] But we continue to have Adverse drug reaction (ADR) associated with individual component of HAART regimens. In this case, we encountered nevirapine induced drug eruption and hepatotoxicity.

Identification of a single antiretroviral drug as the cause of a drug eruption in a patient infected with HIV-1 is often difficult because monotherapy is rarely used. Although our patient was not re-challenged with nevirapine, the signs and symptoms of this patient were most consistent with nevirapine induced SJS, so we believe that nevirapine was responsible for the SJS. SJS or toxic epidermal necrolysis (TEN) has been reported to occur in 0.3% of patients taking nevirapine within the first 4–6 weeks of treatment.[6] In our patient, the hepatic enzymes were elevated to about 4–5 times the normal values, so we had increasing concerns regarding nevirapine associated hepatotoxicity. Severe hepatic reactions attributed to nevirapine as part of HAART or in post-exposure prophylaxis regimens have been reported. Several reports on the hepatic toxicity of nevirapine show that the abnormalities of liver function tests are reversible after discontinuation of the drug.[7] This was witnessed in our patient too over a period of 3–4 weeks.

Identified risk factors for developing hepatotoxicity with nevirapine are female gender, chronic hepatitis C/B virus coinfection, a CD4 count <250 cells/mm^3^ in women and <400/mm^3^ in men, and abnormal baseline transaminase levels.[8]

Most of the drugs which are available and approved for use in HAART have some or the other adverse effect. Thus, treatment of HIV infection has become a complicated balancing act between the benefits of HIV control and the risks of drug toxicity.

Antiretroviral therapy is becoming not only increasingly effective but also increasingly complex. The many adverse effects associated with therapy may cause symptoms affecting a variety of organ systems. Although current antiretroviral regimens are potent from an antiviral perspective, they often do not succeed because of patient’s poor compliance. To optimize adherence, and hence efficacy, clinicians must focus on limiting the adverse effects whenever and wherever possible and distinguishing those that are self-limiting from those that are potentially serious.

In the present case, the other concomitant medication the patient was receiving was stavudine (30 mg) and lamivudine (150 mg) once daily. Both these drugs do not have an obvious association with SJS. According to Hartwig scale, the case was categorized as severe and preventability of the case was probable as per modified Schumock and Thorton scale. The causality assessment of SJS with nevirapine using Naranjo’s Causality Assessment Scale showed a score of 7. WHO Uppsala Monitoring Centre (UMC) Causality Assessment Criteria also indicated a probable association with nevirapine.[9,10]

A strict vigilance on part of treating physician for the initial 2 months at least is necessary to prevent such adverse events. Nevirapine should always be started as lead-in dose (200 mg OD for the first 14 days and then 200 mg BD) to sensitize the system gradually and to avoid serious ADR. The more recent trend is application of Principles of Pharmacogenomics by which we define the particular population or a person who responds differentially to a particular drug, based on their diverse genetic make-up. This has a definite role in high precision multidrug prescription and hence gives a better outcome with fewer side effects.
